# Three New Species of Mytilinidioid Fungi (Dothideomycetes, Ascomycota) from Mexico

**DOI:** 10.3390/jof10100725

**Published:** 2024-10-18

**Authors:** Tania Raymundo, César R. Martínez-González, Michelle Martínez-Pineda, Aurora Cobos-Villagrán, Isabel Ramírez-Rosales, Ricardo Valenzuela

**Affiliations:** 1 Instituto Politécnico Nacional, Escuela Nacional de Ciencias Biológicas, Departamento de Botánica, Laboratorio de Micología, Prolongación de Carpio and Plan de Ayala s.n., Col. Santo Tomás, Alcaldía Miguel Hidalgo, Ciudad de México 11340, Mexico; traymundoo@ipn.mx (T.R.); mmartinezpin@ipn.mx (M.M.-P.); cobos.fungi@gmail.com (A.C.-V.); 2 Departamento de Fitotecnia, Instituto de Horticultura, Universidad Autónoma Chapingo, km 38.5 Carretera Federal México-Texcoco, Texcoco 56230, Estado de México, Mexico; cesar.ramiro.mg@gmail.com; 3 Département de biologie, Faculté des sciences, Université de Sherbrooke, Campus Principale, Sherbrooke, QC J1K 2R1, Canada; ilustrobiologia@gmail.com

**Keywords:** *Acacia californica* subsp. *pringlei*, Hysteriaceae, *Liquidambar styracyphlua*, Mytilinidiaceae, *Pinus patula*, phylogeny, taxonomy

## Abstract

Mytilinidioid fungi are conchiform in nature, with the appearance of bivalve shells or wedge-shaped, rigid, brittle, and carbonaceous hysterothecia growing on the bark of gymnosperms or angiosperms. Based on their morphological characteristics and molecular markers (*ITS* and *LSU*), this study describes three new species of mytilinidioid fungi: *Ericboehmia mexicana* of the family Hysteriaceae of the order Hysteriales and *Lophium pinicola* and *Mytilinidion mexicanum* of the family Mytilinidiaceae of the order Mytilinidiales. The first species grows on *Liquidambar styracyphlua*, the second species grows on *Pinus patula*, and the third species grows on *Acacia californica* subsp. *pringlei*. The specimens studied were deposited in the ENCB Herbarium.

## 1. Introduction

Mytilinidioid fungi are conchiform in nature, with the appearance of bivalve shells or wedge-shaped, rigid, brittle, or carbonaceous hysterothecia. Etymologically, the name of these fungi refers to *Mytilus*, a genus of mussels [1]. These fungi are polyphyletic and classified in the families Hysteriaceae of Hysteriales and Mytilinidiaceae of Mytilinidiales, in the subclass Pleosporomycetidae of the class Dothideomycetes. This group is characterized by being globoid- to obovoid-shaped or strongly laterally compressed, with erect, oyster-shaped hysterothecia standing on edge and lateral walls that are more or less connivant and extended vertically, along with a prominent longitudinal keel or cristate apex and a thin-walled, sclero parenchymatous the genes or lack peridium [2]. Commonly distributed in temperate environments, mytilinidioid fungi are found in association with the wood, bark, resin, cones, scales, needles, seeds, and roots of gymnosperms and angiosperms [2]. In Mexico, only *Mytilinidion mytilinellum* (Fr.) H. Zogg has been recorded, growing on *Pinus pseudostrobus* Lindl. [3]. The objective of this study was to describe three new species of mytilinidioid fungi: one in the family Hysteriaceae (*Ericboehmia mexicana*) and two in the family Mytilinidiaceae (*Lophium pinicola* and *Mytilinidion mexicanum*).

## 2. Materials and Methods

### 2.1. Study Zone

**Figure 1:**
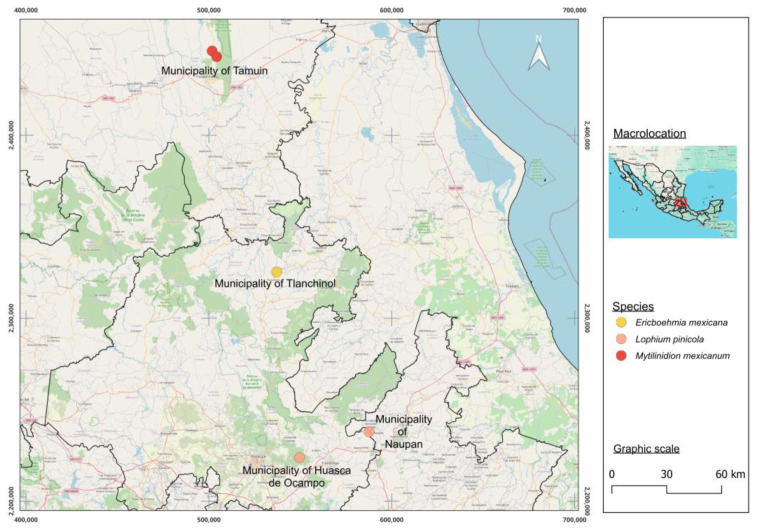
Localities of new species of mytilinidioid fungi in Mexico.

The *Ericboehmia mexicana* specimens were collected from a tropical montane cloud forest in Sierra Alta Hidalguense at Temazate in the municipality of Tlanchinol, Hidalgo State, Mexico, in 2018 (Figure 1). The *Lophium pinicola* specimens were collected from a *Pinus*-*Quercus* forest in Rancho Santa Elena in the municipality of Huasca de Ocampo, Hidalgo State, in 2023 and from the municipality of Naupan, Puebla State, in 2016. The *Mytilinidion mexicanum* specimens were collected from a tropical, dry forest in Las Grutas in the municipality of Tamuín, Biosphere Reserve Sierra del Abra Tanchipa, San Luis Potosí State, in 2023. The specimens were deposited in the fungi collection “Gastón Guzmán Huerta” at the Herbarium of the Escuela Nacional de Ciencias Biológicas (ENCB) of the Instituto Politécnico Nacional.

### 2.2. Morphological Examination

The macroscopic characteristics of the samples are based on descriptions by Boehm et al. (2009a,b) [2,4] and Gardiennet et al. (2019) [5]. The ascomata were measured with a Leica S9-E stereoscopic microscope (Leica Microsystems, Wetzlar, Germany). Subsequently, cross-sections of the ascoma were taken and mounted on slides; then, 70% ethanol was added to hydrate and eliminate air bubbles from the tissues, and 5% potassium hydroxide (KOH) was added to soften and clarify them. The tissues were observed using an Olympus CX3 microscope (Olympus Corporation, Tokyo, Japan) at magnifications of up to 1000×. Images of the structures with taxonomic importance were captured using a Sony DSC-WX350 digital camera (Sony Group Corporation, Tokyo, Japan). The meanings of the taxonomic terms are based on those by Ulloa and Hanlin (2012) [6].

### 2.3. Drawing Techniques

The ascomata were drawn using graphite pencils on 270 g/m^2^ and 27.9 × 35.6 cm Bristol paper. Three-dimensional models were mounted and exposed at 45° angles, illuminating the upper left corners, as indicated by scientific illustration convention. The contrast between darkness and lightness and the harmony of the compositions were also considered to obtain the three-dimensional shapes of the ascomata, along with their textures. The spatial distribution of the mytilinidioid fungi was also represented for each species described as new, and the morphological characteristics of the ascomata were highlighted.

### 2.4. Extraction, Amplification, and Sequencing of DNA

Genomic DNA was extracted from herbarium voucher specimens using the CTAB method [7] and was quantified with a Nanodrop 2000c (Thermo, Waltham, MA, USA). Dilutions of each sample at 20 ng were prepared to amplify two regions of nuclear ribosomal DNA (*nrDNA*), internal transcribed spacer rDNA-ITS1 5.8S rDNA-ITS2 (*ITS*; primers ITS5-ITS4) [8], and ribosomal large subunit-coding DNA (28S rRNA; primers LROR-LR3) [9]. The reaction mixture for PCR was prepared in a final volume of 13 μL, containing a buffer of the enzyme 1× Taq DNA polymerase, 0.8 mM of deoxinucleoside triphosphate (0.2 mM of each), 100 ng of DNA, 20 pmol of each primer, and 2 units of GoTaq DNA (Promega, Madison, WI, USA). PCR amplification conditions were 3 min at 94 °C, followed by 35 cycles of 95 °C for 30 s, 55 °C for 1 min, and 72 °C for 1 min, with a final extension at 72 °C for 10 min for *ITS* and *LSU*. All PCR reactions were undertaken in a Peltier Thermal Cycler PTC-200 (BIORAD, Mexico city, Mexico ). The PCR products were verified via agarose gel electrophoresis. The gels were run for 1 h at 95 V cm^−3^ in 1.5% agarose and 1× TAE buffer (Tris Acetate-EDTA). The gel was stained with GelRed (Biotium, Fremont, CA, USA), and the bands were visualized in an Infinity 3000 transilluminator (Vilber Lourmat, Eberhardzell, Germany). The amplified products were purified using the ExoSAP Purification kit (Affymetrix, Santa Clara, CA, USA), following the manufacturer’s instructions. They were then quantified and prepared for the sequence reaction using BigDye Terminator v.3.1 (Applied Biosystem, Waltham, MA, USA). These products were sequenced in both directions using the Applied Biosystem model 3730XL (Applied BioSystems, Waltham, MA, USA). The sequences of both strands of each gene were analyzed, edited, and assembled using BioEdit version 7.0.5 [10] to generate consensus sequences. These consensus sequences were compared to those deposited in GenBank at the National Center for Biotechnology Information (NCBI) using the BLASTN 2.2.19 tool [11].

### 2.5. Phylogenetic Methods

The newly generated sequences were deposited in GenBank http://www.ncbi.nlm.nih.gov/genbank/ (accessed on 24 July 2024) including one voucher specimen of *Ericboehmia mexicana* with *LSU*, four voucher specimens of *Lophium pinicola* with *ITS* and *LSU*, and one voucher specimen of *Mytilinidion mexicanum* with *ITS* and *LSU* markers.

The sequence data, retrieved from GenBank for previous studies on *Ericboehmia*, are listed in Table 1. The sequences were aligned using as an external group to *Dendrographa decolorans* (Turner & Borrer) Ertz & Tehler (HQ454610). For the Mytilinidiales, the sequences were subjected to standard BLAST searches in GenBank to determine the primary identities of the fungal isolates of Mytilinidiales, as listed in Table 2. *Glonium circumserpens* (Nyl.) Kantvilas & Coppins and *Cenococcum geophilum* Fr. [12] were used as the outgroup. The *LSU* region of *Ericboehmia* and each region of the Mytilinidiales were independently aligned using the online version of MAFFT v. 7 [13,14,15]. The alignment was revised in PhyDE v. 10.0 [16], followed by minor manual adjustments to ensure character homology between taxa. Two matrices were generated: the *Ericboehmia* dataset included *LSU* sequences from 39 specimens, representing 26 taxa (650 characters), and the Mytilinidiales dataset included *ITS* sequences from 19 specimens, with 9 taxa (685 characters), and *LSU* sequences from 22 specimens, with 12 taxa (585 characters). Two partition schemes were established: one for *ITS* and one for *LSU*, which were established using the option to minimize the stop codons with Mesquite v3.2 [17]. 

In both datasets, the data were analyzed using maximum parsimony (MP), maximum likelihood (ML), and Bayesian inference (BI). Maximum parsimony analyses were carried out in PAUP* 4.0b10 [18] using the heuristic search mode, 1000 random starting replicates, and TBR branch swapping, with MULTREES and Collapse on. Bootstrap values were estimated using 1000 bootstrap replicates under the heuristic search mode, each with 100 random starting replicates. Maximum likelihood analyses were carried out in RAxML v. 8.2.10 [19] with a GTR + G model of a nucleotide substitution. To assess the branch support, 10,000 rapid bootstrap replicates were run using the GTRGAMMA model. Bayesian inference analyses were carried out in MrBayes v. 3.2.6 x64 [20] with four chains. The best evolutionary model for alignment was sought using PartitionFinder [21,22,23]. The information block for the matrix included two simultaneous runs of Montecarlo chains, with the temperature set to 0.2 and sampling of 10 million generations (standard deviation ≤ 0.1). Chain convergence was visualized in Tracer v.1.6 [24]. The remaining trees were used to calculate a 50% majority rule consensus topology and posterior probabilities (PPs). The trees were visualized and optimized in FigTree v. 1.1.4 [25] and were edited in Adobe Illustrator (Adobe Systems, Inc., San Jose, CA, USA).

**Table 1:** GenBank accession numbers for the taxa used in the phylogenetic analysis of *Ericboehmia*. The sequences generated for this study are in bold.

Taxon	Culture Accession No. or Voucher Specimen	GenBank Accession No.	Country of Origin of the Sequence	Source
		*LSU*		
*Dendrographa decolorans*	Ertz 14063	HQ454610	Belgium	[26]
*Ericboehmia centramura*	MFLUCC 12-0808	KM272256	Thailand	[27]
*Ericboehmia curtisii*	CBS:198.34	FJ161176	USA	[2,4]
*Ericboehmia curtisii*	CBS:198.34	MH866967	USA	[5]
*Ericboehmia doimaeensis*	MFLUCC 16-0329	MH535894	Thailand	[28]
***Ericboehmia mexicana***	**T. Raymundo 7609**	**PP575996**	**Mexico**	**This study**
*Ericboehmia saulensis*	AG18089	MN338581	French Guiana	[5]
*Ericboehmia thailandica*	MFLUCC 16-0338	MH535895	Thailand	[28]
*Gloniopsis calami*	MFLUCC 15-0739	NG_059715	Thailand	[29]
*Gloniopsis fluctiformis*	C419	MK348005	Thailand	[30]
*Gloniopsis leucaenae*	C289	MK347967	Thailand	[30]
*Gloniopsis praelonga*	CBS:123337	FJ161195	USA	[4]
*Hysterium angustatum*	CMW:20409	FJ161194	USA	[2,4]
*Hysterium angustatum*	MFLUCC 16-0623	MH535893	Italy	[28]
*Hysterium angustatum*	GKM5211	GQ221906	New Zealand	[31]
*Hysterium barrianum*	ANM1442	GQ221884	USA	[31]
*Hysterium barrianum*	ANM1495	GQ221885	USA	[31]
*Hysterium pulicare*	CBS:123377	FJ161201	USA	[4]
*Hysterium pulicare*	CBS:240.34	MH866998	USA	[32]
*Hysterium vermiforme*	GKM1234	GQ221897	Kenya	[31]
*Hysterobrevium constrictum*	GKM426N	GQ221901	Kenya	[31]
*Hysterobrevium mori*	CBS:123564	FJ161198	USA	[4]
*Hysterobrevium mori*	CBS:123335	FJ161202	USA	[4]
*Hysterobrevium mori*	CBS:123563	FJ161196	USA	[4]
*Hysterobrevium rosae*	MFUCC 14-0551	MH535897	Italy	[28]
*Hysterobrevium rosae*	MFUCC 14-0552	MH535898	Italy	[28]
*Hysterobrevium smilacis*	CBS:114601	FJ161174	Sweden	[2]
*Hysterobrevium smilacis*	CBS:200.34	MH866968	USA	[2]
*Hysterodifractum partisporum*	HUEFS:42865	KF914916	Brazil	[33]
*Hysterodifractum partisporum*	HUEFS 42865	NG_060652	Brazil	[33]
*Ostreichnion sassafras*	CBS:322.34	FJ161188	USA	[2]
*Ostreichnion sassafras*	CBS:322.34	MH867054	USA	[32]
*Psiloglonium araucanum*	CBS:112412	FJ161172	South Africa	[4]
*Psiloglonium araucanum*	CMW:18760	FJ161192	South Africa	[4]
*Psiloglonium clavisporum*	CBS:123338	FJ161197	USA	[4]
*Psiloglonium clavisporum*	CBS:123341	FJ161206	USA	[4]
*Rhytidhysteron hysterinum*	EB 0351	GU397350	France	[4]
*Rhytidhysteron neorufulum*	MFLUCC 13-0221	KU377567	Thailand	[33]
*Rhytidhysteron rufulum*	GKM361A	GQ221893	Kenya	[31]

**Table 2:** GenBank accession numbers for the taxa used in the phylogenetic analyses of *Lophium* and *Mytilinidion*. The sequences generated for this study are in bold.

	Voucher	Country	Isolation Source	GenBank Accession No.		Source
				*ITS*	*LSU*	
*Cenococcum geophilum*	1-17-2	USA	-----	-----	JN860135	[34]
*Glonium circumserpens*	CBS 123343	Australia	Saxicolous	-----	FJ161200	[2]
*Lophium arboricola*	CBS 758.71 Type	United Kingdom	*Larix decidua*	NR153447	NG064094	[12]
*Lophium arboricola*	NW-FVA 6260	Germany	*Acer pseudoplatanus*	ON710911	-----	[12]
*Lophium arboricola*	ZK52b/08	Czech Republic	*Picea abies*	FR837917	FR837917	[12]
*Lophium arboricola*	P98	Poland	*Picea abies*	OR754901	OR754923	[12]
*Lophium arboricola*	P99	Poland	*Abies alba*	OR754902	OR754924	[12]
*Lophium arboricola*	CBS 102826	Spain	On dung	KU705825	KU705842	[12]
*Lophium mytilinum*	CBS 123344	USA	*Pinus strobus*	-----	FJ161203	[2]
*Lophium mytilinum*	CBS 269.34	USA	*Pinus* sp.	EF596817	EF596817	[35]
*Lophium mytilinum*	CBS 114111	Sweden	*Pinus sylvestris*	EF596819	EF596819	[2]
***Lophium pinicola***	**T. Raymundo 9516 Type**	**Mexico**	*Pinus patula*	**PQ149439**	**PQ151434**	**This study**
***Lophium pinicola***	**T. Raymundo** **6015**	**Mexico**	***Pinus patula***	**PQ149440**	**PQ151435**	**This study**
***Lophium pinicola***	**R. Valenzuela 18065**	**Mexico**	***Pinus patula***	**PQ149441**	**PQ151436**	**This study**
***Lophium pinicola***	**Mart.-Pineda 2300**	**Mexico**	***Pinus patula***	**PQ149442**	**PQ151437**	**This study**
*Lophium zalerioides*	MFLUCC 14-0417	Italy	-----	MF621583	MF621587	[36]
***Mytilinidion mexicanum***	**T. Raymundo 9300 Type**	**Mexico**	***Acacia californica*** **subsp. *pringlei***	**PQ149443**	**PQ151438**	**This study**
*Mytilinidion resinicola*	CBS 304.34 Type	USA	*Larix laricina*	MH855535	MH867038	[2]
*Mytilinidion rhenanum*	EB 0341	France	-----	-----	GU323207	[35]
*Mytilinidion scolecosporum*	CBS 305.34 Type	USA	*Pinus strobus*	NR160069	NG057808	[2]
*Pseudocamaropycnis pini*	CBS 115589 Type	China	Leaf of *Pinus elliotii*	KU728518	KU728557	[37]
*Slimacomyces isiolus*	FP1465	Japan	-----	AB597207	AB597217	[12]
*Slimacomyces isiolus*	P10436	Japan	-----	AB597213	AB597220	[12]

## 3. Results

### 3.1. Phylogenetic Analyses

**Figure 2:**
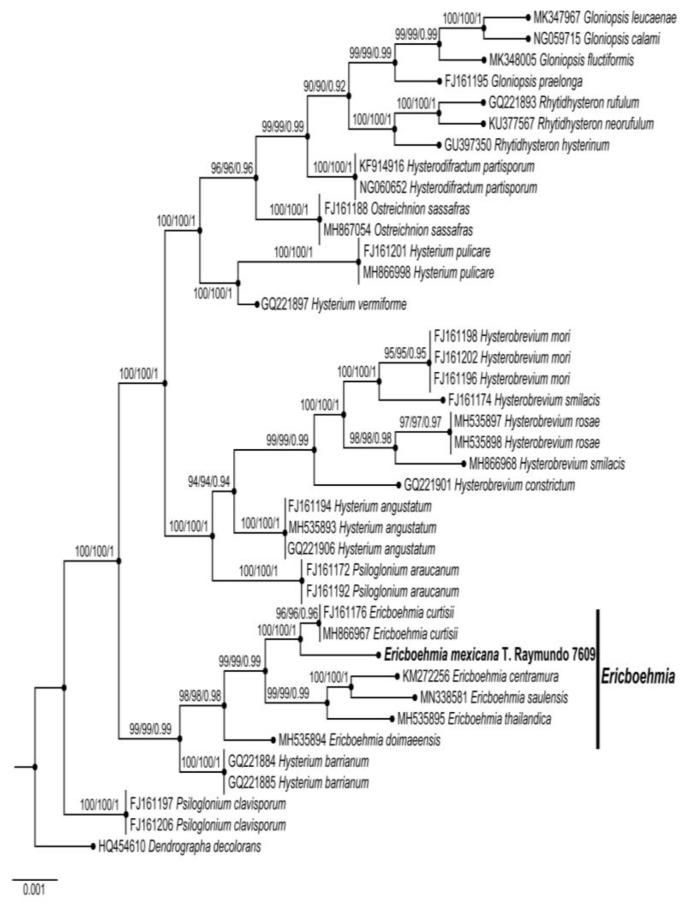
Phylogenetic tree reconstructed from the alignment of *LSU* nucleotide sequences by Bayesian inference. For each node, the following values are provided: maximum parsimony bootstrap (%)/maximum likelihood bootstrap (%) and posterior confidence (*p*-value). The new species, *Ericboehmia mexicana*, is shown in bold.

We successfully amplified and sequenced the *LSU* region from one Mexican specimen of *Ericboehmia*. After the incorporation of additional sequences downloaded from GenBank (Table 1), the aligned *LSU* dataset included 720 characters (including gaps), of which 457 were conserved sites, 157 were variable sites, and 106 were parsimony informative sites. The three phylogenetic analyses (MP, ML, and BI) of the *LSU* dataset recovered similar topologies (Figure 2). No significant conflicts (bootstrap value > 70%) were detected among the topologies obtained via the separate phylogenetic analyses. The parsimony analysis of the alignment found 942 trees of 210 steps (CI = 0.3045, HI = 0.1084, RI = 0.5176, and RC = 0.5095). The best RAxML tree, with a final likelihood value of –30,521.086301 of a new species based on morphological characters and phylogenetic analysis of *LSU* (GenBank accession number PP575996) sequences, is presented in Figure 2. The matrix had 1010 distinct alignment patterns, with 5.14% undetermined characters or gaps. The estimated base frequencies were as follows: A = 0.106245, C = 0.272982, G = 0.128304, and T = 0.280642. The substitution rates were as follows: AC = 1.075210, AG = 1.284501, AT = 1.182456, CG = 1.007215, CT = 5.348215, and GT = 1.000000. The gamma distribution shape parameter was α = 0.002754. In the Bayesian analysis, the standard deviation between the chains stabilized at 0.00002 after 3 million generations. No significant changes in the tree topology trace or cumulative split frequencies of the selected nodes were observed after about 0.25 million generations, which were discarded as 25% burn-in. The phylogenetic analysis of *LSU* showed that *Ericboehmia mexicana* is monophyletic, with strong support (BS = 100%, BS = 100%, and BI p = 1), and is distinctive from other species. The distinct morphology and *LSU* sequences provide evidence that this is a new species.

**Figure 3:**
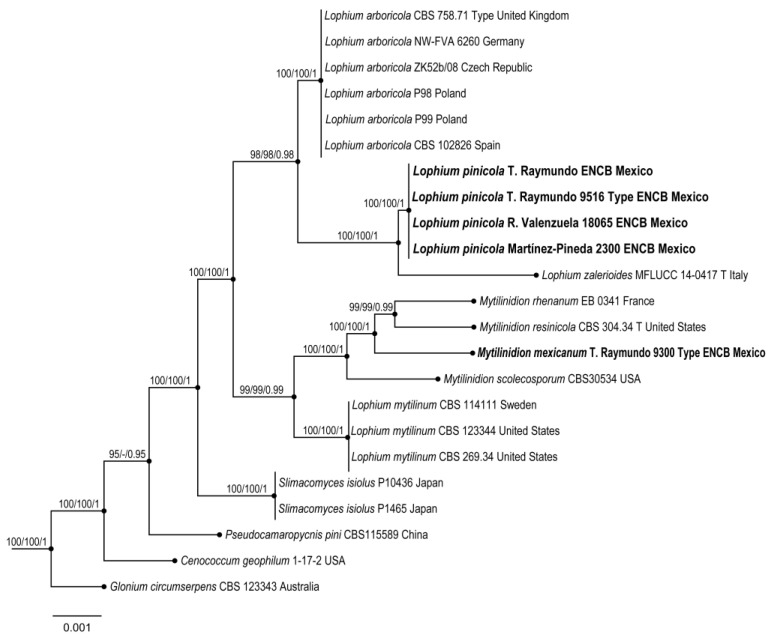
Maximum likelihood phylogeny based on concatenated *ITS* and *LSU* sequence alignment. Maximum parsimony and Bayesian analyses recovered identical topologies with respect to the relationships among the main clades of members of the order Mytilinidiales. For each node, the following values are provided: maximum parsimony bootstrap (%)/maximum likelihood bootstrap (%) and posterior confidence (*p*-value). The scale bar represents the expected number of nucleotide substitutions per site. The new species, *Lophium pinicola* and *Mytilinidion mexicanum*, are shown in bold.

The Mytilinidiales dataset of combined *ITS* and *LSU* markers comprised 23 specimens in 12 taxa, with 1270 characters (including gaps). The three phylogenetic analyses (MP, ML, and BI) of the dataset recovered similar topologies (Figure 3). No significant conflicts (bootstrap value > 80%) were detected among the topologies obtained via the separate phylogenetic analyses. The parsimony analysis of the alignment found 856 trees from 245 steps (CI = 0.2560, HI = 0.1002, RI = 0.2812, and RC = 0.1058). The best RAxML tree, with a final likelihood value of −24,012.573014, is presented in Figure 3. The matrix had 985 distinct alignment patterns, with 3.02% undetermined characters or gaps. The estimated base frequencies were as follows: A = 0.102410, C = 0.108420, G = 0.100814, and T = 0.100578. The substitution rates were as follows: AC = 1.000475, AG = 1.001835, AT = 1.000824, CG = 1.000221, CT = 4.000421, and GT = 1.100000. The gamma distribution shape parameter was α = 0.002014. In the Bayesian analysis, the standard deviation between the chains stabilized at 0.001 after 4.5 million generations. No significant changes in the tree topology trace or cumulative split frequencies of the selected nodes were observed after about 0.25 million generations, which were discarded as 25% burn-in. The analyses produced a phylogenetic tree in which *Lophium pinicola* and *Mytilinidion mexicanum* were shown as monophyletic groups (BS = 100%, BS = 100%, and BI p = 1).

### 3.2. Taxonomy

***Ericboehmia mexicana*** Cobos-Villagrán, Raymundo & R. Valenz. sp. nov.

Mycobank: MB855225.

**Figure 4:**
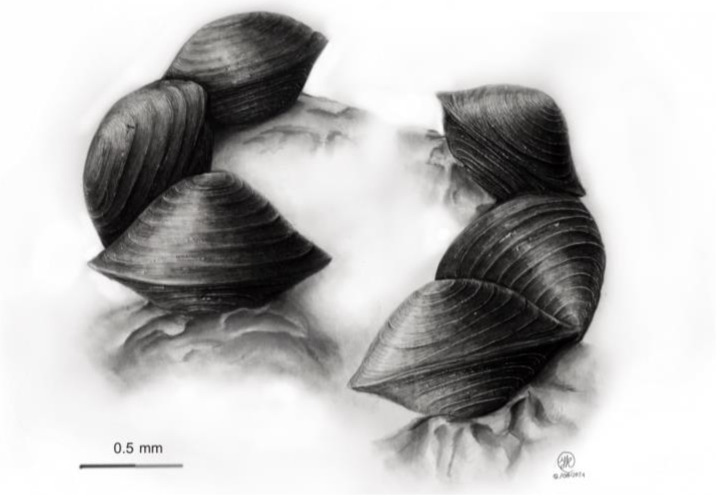
Drawing of *Ericboehmia mexicana* Cobos-Villagrán, Raymundo & R. Valenz., showing details of the shape, texture, and distribution of the ascomata.

**Figure 5:**
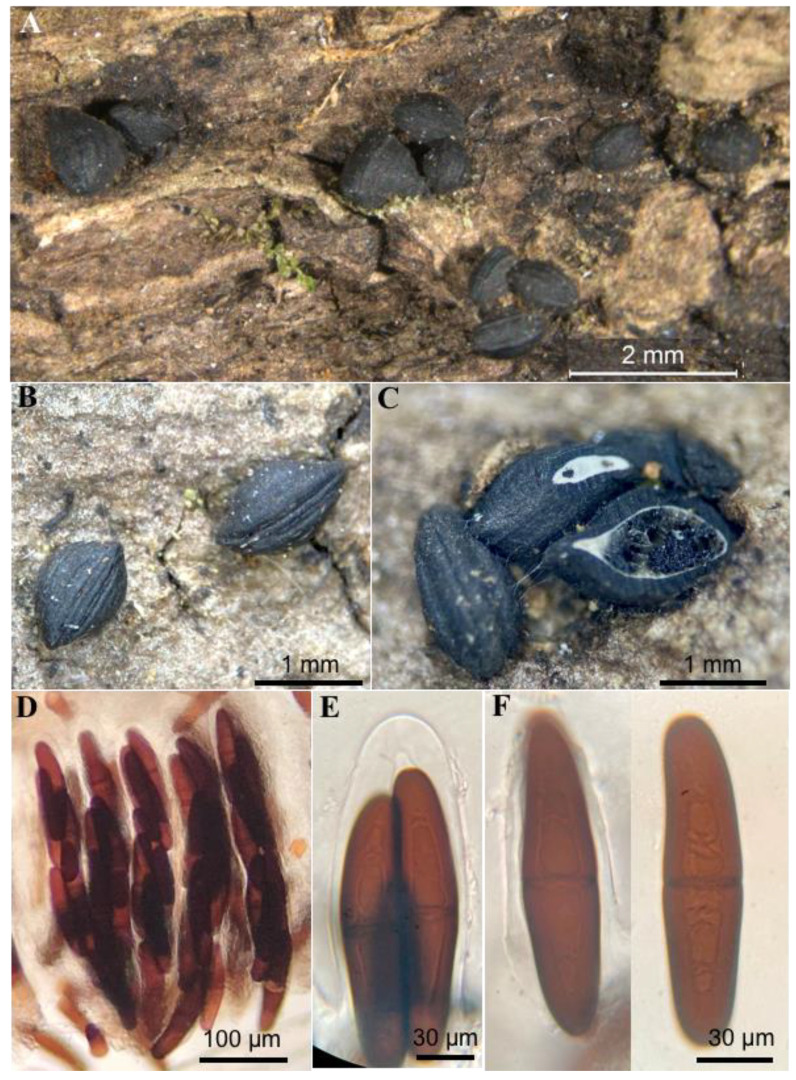
*Ericboehmia mexicana* Cobos-Villagrán, Raymundo & R. Valenz.: (**A**–**C**) ascomata; (**D**) hymenium; (**E**) ascus apex with ascospores; (**F**) ascospores.

Figure 1, Figure 2, Figure 4 and Figure 5.

Diagnosis: This species differs from *Ericboehmia* species in the large size of asci (336–) 345–439 (–447) × 60–90 μm and the size of ascospores (120–) 132–150 × (25–) 29–32 (–40) growing on *Liquidambar styraciflua* L.

Type: Mexico, Hidalgo State, Tlanchinol municipality, 168 km Pachuca–Tampico Road, El Temazate, 21°01′40″ N, 98°38′33″ W, 1500 m, 31 May 2018, T. Raymundo 7609 (ENCB, Holotype).

GenBank: LSU: PP575996.

Etymology: The epithet refers to the name of the country where the species was collected.

Ascomata 1200–1600 μm long, 1000–1100 μm wide, and 800–1000 μm high (*n* = 10), shell-shaped, with a laterally flattened and narrow base and a convex upper ridge, with a longitudinal slit and grooves along its longitudinal axis. gregarius in small, scattered clusters, erumpent to shallow, carbonaceous, and dull black to slightly shiny. Peridium 20–150 × 115–120 μm thick, with a thin upper part of up to 20 μm and a thickened base of up to 150 μm, composed of sclerenchymatous cells of textura globulosa to textura angularis. Pseudoparaphyses 1.5–2 μm wide, branched, hyaline, without septa, and embedded in a gelatinous matrix. Asci (336–) 345–439 (–447) × 60–90 μm, bitunicate, 8-spored, biseriate, cylindrical to clavate, and hyaline. Ascospores (120–) 132–150 × (25–) 29–32 (–40) μm (*n* = 30), pale brown, reddish brown to dark brown, slightly fusiform when young, becoming oblong, smooth, with a single central transverse septum.

Distribution and ecology: This species grows on the bark of fallen logs of *Liquidambar styraciflua* in a tropical montane cloud forest. It is only known from the type locality.

Specimens examined: Mexico, Hidalgo State, Tlanchinol municipality, 168 km Pachuca–Tampico Road, El Temazate, 21°01′40″ N, 98°38′33″ W, 1500 m, 31 May 2018, R. Valenzuela 18309 (ENCB), A. Cobos-Villagrán 1720 (ENCB), S. Cuevas-Pérez 20 (ENCB), A. Ramírez-Chavarín 20 (ENCB.

Taxonomic notes: *Ericboehmia mexicana* is distinguished from the other species of the genus by having larger ascospores. The ascomata are morphologically similar to those of *E. beejakoshae* (M. Niranjan & V.V. Sarma) Gardiennet, Lechat & J. Fourn. and *E. appendiculata* (R.M. Sánchez & Bianchin.) Gardiennet, Lechat & J. Fourn., and both have smaller ascospores, the first being 42.5–57.5 × 10.5–13.7 μm and from Andaman and Nicobar Islands, India [38], and the second being 58–88 × 15–28 μm and from Argentina [39]. Phylogenetically, *E. mexicana* is closely related to *E. curtisii* (Duby) Gardiennet, Lechat & J. Fourn., but *E. curtisii* is distinguished by having smaller ascomata (0.3–0.7 × 0.1–0.3 × 0.2–0.3 mm vs. 1.2–1.6 × 1–1.1 × 0.8–1 mm and smaller and transversally multiseptated ascospores ((45–) 62–82 × (10–) 12–15 (–17) µm vs. (120–) 132–150 × (25–) 29–32 (–40) µm) [4].

***Lophium pinicola*** Raymundo, Mart.-Pineda & R. Valenz. sp. nov.

MycoBank: MB855226.

**Figure 6:**
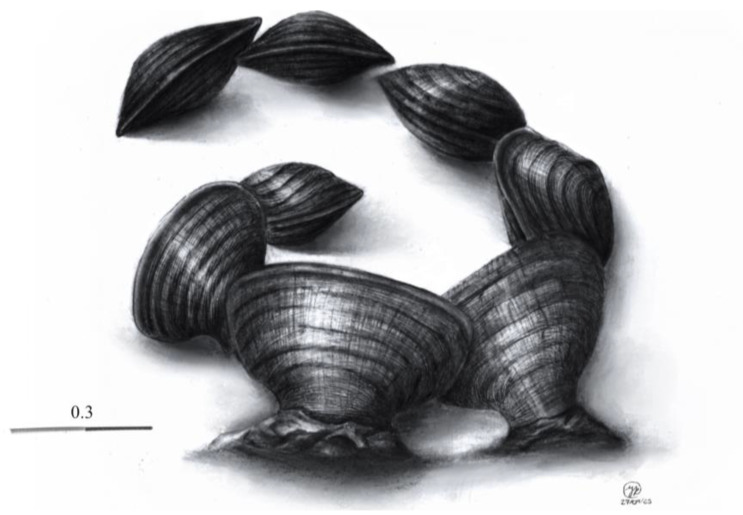
Drawing of *Lophium pinicola* (Raymundo, Mart.-Pineda, and R. Valenz), showing details of the shape, texture, and distribution of the ascomata.

**Figure 7:**
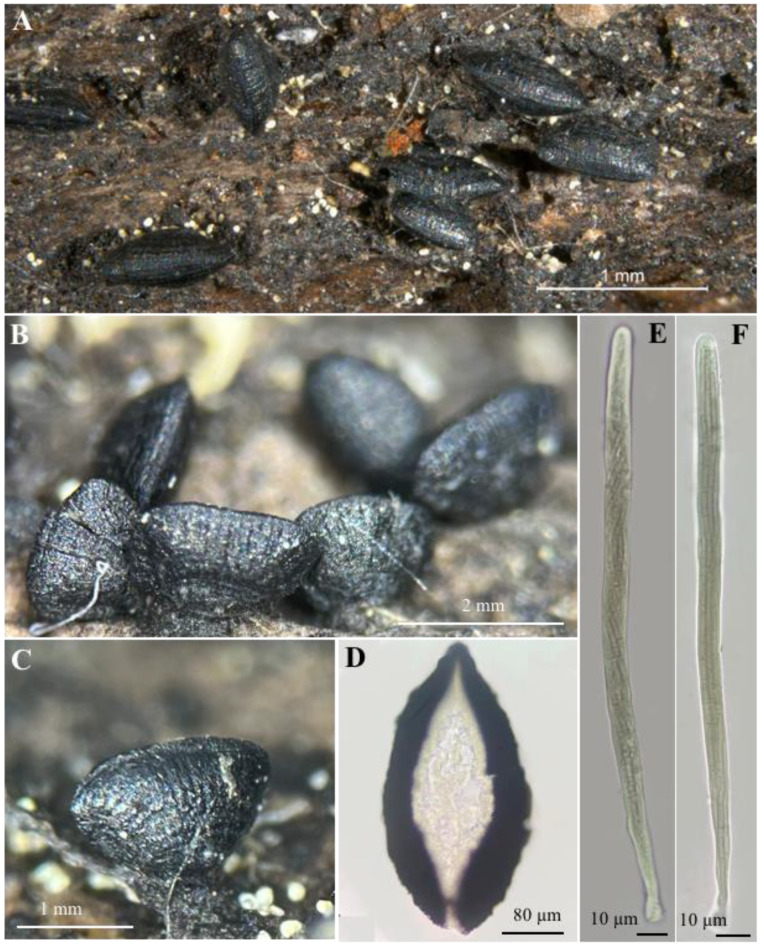
*Lophium pinicola* (Raymundo, Mart.-Pineda, and R. Valenz): (**A**–**C**) ascomata; (**D**) hymenium; (**E**,**F**) asci and ascospores.

Figure 1, Figure 3, Figure 6 and Figure 7.

Diagnosis: This species differs from *Lophium mytilinidion* in that the ascospores are 198–200 × 1–2 μm, spirally arranged, and grow on *Pinus patula* Schiede ex Aschltdl &Cham.

Type: Mexico, Hidalgo State, Huasca de Ocampo municipality, Rancho Santa Elena Reserve, 20°06′39″ N, 98°31′32″ W, 2617 m, 16 October 2023, T. Raymundo 9516 (ENCB, Holotype).

GenBank: ITS: PQ149439, PQ149440, PQ149441, and PQ149442; LSU: PQ151434, PQ151435, PQ151436, and PQ151437.

Etymology: The epithet refers to the host *Pinus patula* on which the species was found.

Ascomata 750–2000 μm long, 300–400 μm wide, and 160–200 μm high, mytilinidioid, conchiform, dimidiate, black, shiny and dull with age, with a horizontally and vertically striated surface. Peridiumthin and scleroparenchimatous. Hamathecium has trabeculate pseudoparaphyses, up to 1 μm, filiform and hyaline. Asci 190–210 × 7–8 μm, bitunicate, cylindrical, hyaline, and eight-spored. Ascospores 198–200 × 1–2 μm, filiform, fasciculate, spirally arranged, hyaline to yellowish, multiseptate, smooth, and thin-walled.

Habitat: This species grows on fallen logs of *Pinus patula* in *Pinus-Quercus* forests.

Additional specimens: Mexico, Hidalgo State, Huasca de Ocampo municipality, Rancho Santa Elena Reserve, LN 20°06′39″ LW 98°31′32″, 2617 m, 16 October 2023, R. Valenzuela 18065 (ENCB). Puebla State, Naupan municipality, 8 km Tejocotal–Naupan Road, 8 May 2016, R. Valenzuela 16515 (ENCB), T. Raymundo 6015 (ENCB), M. Mtz-Pineda 2300 (ENCB).

Taxonomic notes: *Lophium pinicola* is distinguished from the other species in the genus by the combination of the following characteristics: the size of the ascomata, the host, and the size and spiral arrangement of the ascospores within the asci. *Lophium mytilinum* is similar in that it grows on *Pinus* spp. but differs in terms of its ascospores, which are parallel in the asci and slightly larger ((130–) 170–250 (–300) × 1–2 μm), with a cosmopolitan distribution [4]. *Lophium elegans* H. Zoggs is similar in that it has ascospores that are spirally arranged in the asci but differs in that it has larger ascospores ((200–) 260–280 (–300) × 2 μm) and grows on *Juniperus* in Europe [4]. Phylogenetically, *L. pinicola* is closely related to *L. zalerioides* Jun F. Li, Phook., Camporesi & K.D. Hyde but *L. zalerioides* was only found in its asexual morph, growing on fallen cones of *Cupressus glabra* Sudw. in Italy [36].

***Mytilinidion mexicanum*** Raymundo, Mart.-Pineda &R. Valenz sp. nov.

MycoBank: MB855227.

**Figure 8:**
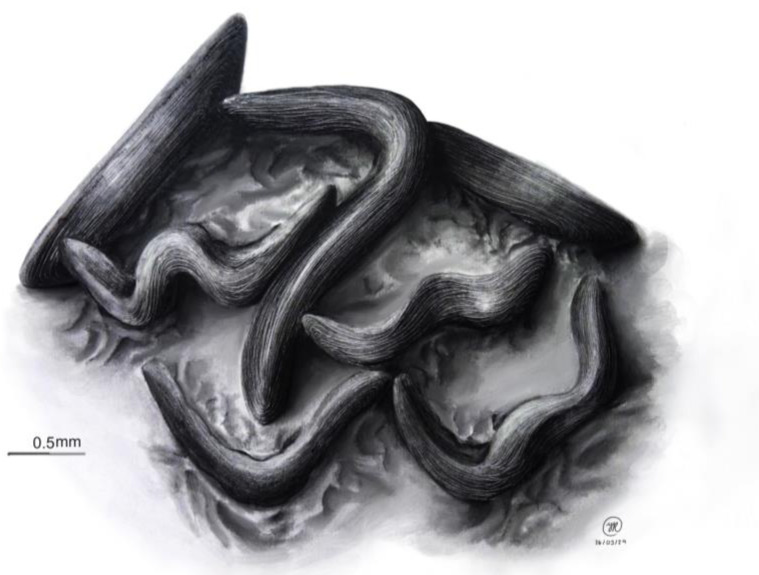
Drawing of *Mytilinidion mexicanum* Raymundo, Mart.-Pineda & R. Valenz., showing details of the shape, texture, and distribution of the ascomata.

**Figure 9:**
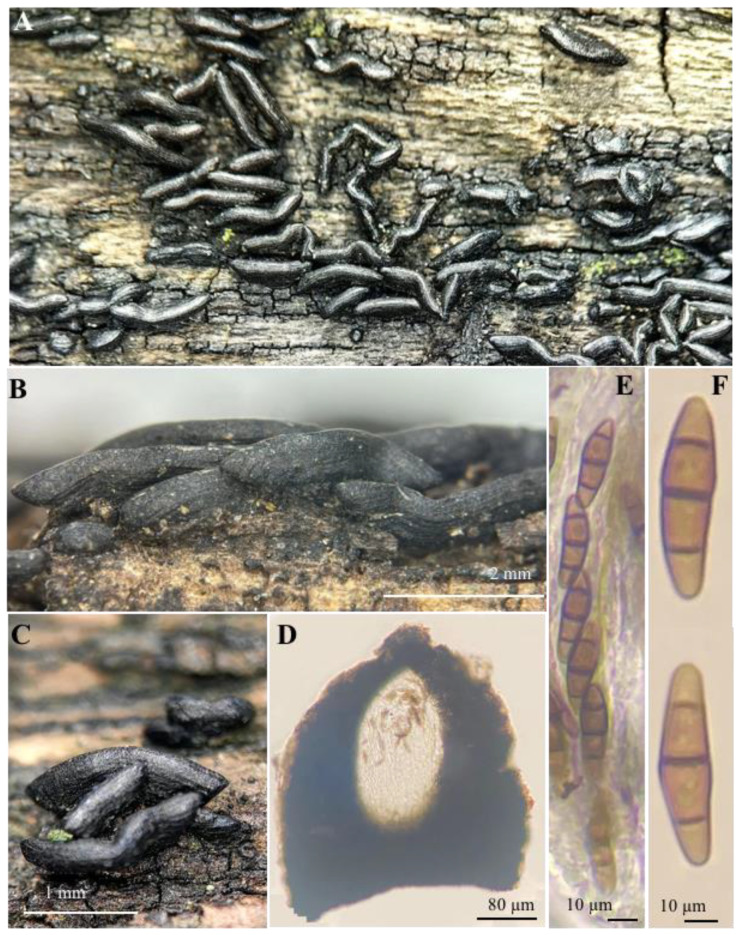
*Mytilinidion mexicanum* Raymundo, Mart.-Pineda & R. Valenz.: (**A**–**C**) ascomata; (**D**) hymenium; (**E**) asci; (**F**) ascospores.

Figure 1, Figure 3, Figure 8 and Figure 9.

Diagnosis: This species differs from *Mytilinidion mytilinellum* in that it has ascospores that are (15–) 17–20 (–22) × 4–5 μm, oblong, fusiform, slightly constricted at the middle septum, pale brown to dark brown, and slightly lighter end cells, as well as having round ends and growing on *Acacia californica* subsp. *pringlei* (Rose) L. Rico.

Type: Mexico, San Luis Potosi State, Tamuin municipality, Biosphere Reserve Sierra del Abra Tanchipa, Ejido Los Sabinos Dos, Las Grutas, 22°07′11″ N, 98°58′58″ W, 24 June 2023, T. Raymundo 9300 (ENCB, Holotype).

GenBank: ITS: PQ149443; LSU: PQ151438.

Etymology: The epithet refers to the country where the species was collected.

Ascomata 750–2000 μm long, 160–180 μm wide, and 200–300 μm high, mytilinidioid, conchiform to elongate, dimidiate to broadly attached to wood, black, and shiny to dull, with a horizontally striated surface. Peridium 97–110 μm thick, carbonaceous, textura globosa, pseudoparenchimatous, and thick-walled, with globose cells. Pseudoparaphyses up to 1 μm in diameter, filiform, branched, anastomosed, septate, and hyaline. Asci 90–95 × 5–6 μm, bitunicate, cylindrical, pediculated, 8-spored, hyaline, and uniseriate. Ascospores (15–) 17–20 (–22) × 4–5 μm, oblong-fusiform, with rounded ends, slightly curved, pale brown to dark brown, slightly lighter in the end cells, 3-septate, slightly constricted at the middle septum, the second largest cell, smooth, and thin-walled.

**Habitat:** Gregarious, growing on the decaying wood of *Acacia californica* subsp. *pringlei* in tropical dry forests.

**Additional specimens:** Mexico, San Luis Potosi State, Tamuin municipality, Biosphere Reserve Sierra del Abra Tanchipa, Ejido Los Sabinos Dos, Las Grutas, 22°07′11″ N, 98°58′58″ W, 24 June 2023, R. Valenzuela 17876 (ENCB); 13 November 2023, T. Raymundo 9676 (ENCB), T. Raymundo 9679 (ENCB), T. Raymundo 9681 (ENCB), and T. Raymundo 9682 (ENCB).

**Taxonomic notes:** *Mytilinidion mexicanum* is distinguished from the other species of the genus in that it has 3-septate, (15–) 17–20 (–22) × 4–5 μm, and oblong-fusiform ascospores and grows on the decaying wood of *Acacia californica* subsp. *pringlei* in tropical dry forests. *Mytilinidion mytilinellum* differs in having (2–) 3 (–5)-septate, (14–) 16–22 (–24) × (2.5–) 3–4 (–5) μm, and elongate ascospores, growing on coniferous hosts, and having a cosmopolitan distribution [4]. *M. tortile* is similar because it has 3-septate, oblong to fusiform, and slightly curved ascospores but differs in that it has smaller ascospores (11–14 × 5–7 μm) and grows on *Larix* and *Juniperus* in Europe [4]. Phylogenetically, *M. mexicanum* is closely related to *M. resinicola* M.L. Lohman and *M. rhenanum* Fuckel, but *M. resinicola* has elliptic to oblong and larger ascospores (24–26 × 8–9 μm), which are deeply constricted at the septa and grow on the resinous bark of *Larix laricina* (Du Roi) K. Koch in the USA [4]. *M. rhenanum* differs in that it has 3–5 (–7)-septate, (24–) 30–42 (–50) × 3–5 μm, and elongate ascospores, which grow on the roots of rotten stumps of *Pinus sylvestris* L. in Germany [4].

## 4. Discussion

Mytilinidioid fungi are a morphological group that has been segregated into the orders Hysteriales and Mytilinidiales; however, this name is used to separate them from other morphological groups of Dothideomycetes. In the present study, phylogenetic analyses of *Ericboehmia mexicana* (Hysteriales), *Lophium pinicola*, and *Mytilinidion mexicanum* (Mytilinidiales) were performed separately. Therefore, they will be discussed separately in this section.

The genus *Ericboehmia* was segregated from *Ostreichnion*, another mytilinidioid fungus, due to their morphological and phylogenetic differences [5]. The septation of the ascospores is the main morphological characteristic that distinguishes *Ostreichnion* from *Ericboehmia*: *Ostreichnion* has muriform ascospores, while *Ericboehmia* has two-celled ascospores with sometimes secondary, more or less developed, distosepta, making them phylogenetically distant [5]. *Ericboehmia mexicana* is a mytilinidioid fungus, with oyster-shaped ascomata, a prominent, longitudinal keel or crested apex, a thin and brittle peridium, and a trabeculated gelatinous matrix. Microscopically, it has bicellular ascospores, characteristic of the genus, but they are larger than those of the other species of *Ericboehmia*. The *Ericboehmia mexicana* samples were collected from *Liquidambar styraciflua* in a tropical montane cloud forest in the neotropical region of Mexico. Seven species of *Ericboehmia* have been recognized in the Index Fungorum (https://www.indexfungorum.org/, accessed on 15 July 2024). Five species are found in tropical regions of South America and Southeast Asia: *Ericboehmia saulensis* Gardiennet, Lechat & J. Fourn., has been reported in French Guiana on dead, corticated twigs of *Caesalpinia pulcherrima* (L.) Swartz; *E*. *centramura* (Senan.) Gardiennet, Lechat & J. Fourn., *E*. *doimaeensis* (Jayasiri & K.D. Hyde) Gardiennet, Lechat & J. Fourn., and *E*. *thailandica* (Jayasiri & K.D. Hyde) Gardiennet, Lechat & J. Fourn. have been found on decaying wood in Thailand; and *E*. *beejakoshae* has been collected from thorny twigs in the Adaman Islands. Two species grow in temperate forests in South America and North America: *E*. *appendiculata* has been collected from the bark of *Nothofagus pumilio* (Poepp. & Endl.) Krasser in Patagonia and Argentina, and *E*. *curtisii* has been found on the branches of *Vitis* in South Carolina, USA [5,27,28,29,30]. The morphological and ecological differences between *E*. *mexicana* and the other species described in the genus, together with phylogenetic analyses, demonstrate that it is a new species.

*Lophium piniciola* and *M. mexicanum* are classified as monophyletic groups in Mytilinidiales, because they form well-supported phylogenetic clades. *Lophium pinicola* is phylogenetically related to *L. arboricola* and *L. zalerioides*; however, both species have only been described in the asexual phase. The first species grows on *Larix decidua* Mill. [40], *Picea abies* (L.) H. Karst., and *Abies alba* Mill. in the Czech Republic [41], England [42], Norway [42], and Poland [12], while it has been recorded on *Acer pseudoplatanus* L. in Germany [43]. The second species has been recorded in Italy, growing on *Cupressus glabra* [36]. The *Lophium pinicola* samples were collected from *Pinus patula* in *Pinus*-*Quercus* forests in the Hidalgo and Puebla States of Mexico. On the other hand, the *Mytilinidion mexicanum* samples were collected from decaying wood of *Acacia californica* subsp. *pringlei* in a tropical dry forest in Biosphere Reserve Sierra del Abra Tanchipa, San Luis Potosí State, Mexico. Morphologically, it is separate from the other species of the genus, because it has 3-septate, oblong-fusiform, and slightly curved ascospores. Phylogenetically, it is close to *M. rhenanum* and *M. resinicola*, but the first species has larger, filiform, and 3–5-septate ascospores and grows on coniferous wood in Norway and Switzerland [4,44], while *M. resinicola* has elliptic to oblong and 3-septate ascospores [4]. Finally, another close species is *M. scolescosporum* M.L. Lohman, which is characterized as having 5–7-septate and 40–50 × 2–2.5 μm ascospores and grows on *Pinus strobus* L. in the USA [45].

## 5. Conclusions

Mytilinidioid fungi are an artificial group of Dothideomycetes, which have been little studied, because they are difficult to find due to their microscopic size. They are characterized as having ascomata with the appearance of bivalve shells and living on coniferous leaves and wood or the resinous cones and rotten wood of angiosperms in temperate and tropical forests. This study was the first to be carried out on mytilinidioid fungi in Mexico and described three new species, with morphological, ecological, and phylogenetic evidence. The new species are *Ericboehmia mexicana*, *Lophium pinicola*, and *Mytilinidion mexicanum*. This study demonstrates the great diversity of mytilinidioid fungi, which are part of the most diverse class in the Phylum Ascomycota, and the lack of studies on them in both temperate and tropical zones. It is necessary to collect samples from unexplored regions and look for them in different types of vegetation to learn about their ecological and geographical distribution in Mexico and the world. Additionally, it is essential to employ more molecular markers to generate more robust phylogenetic trees.

## Data Availability

The original contributions presented in the study are included in the article, further inquiries can be directed to the corresponding author.
